# Pulmonary sarcoid-like granulomatosis induced by aluminum dust: A case report and literature review

**DOI:** 10.3389/fmed.2023.1085716

**Published:** 2023-02-14

**Authors:** Xuqin Du, Lihong Song, Ruie Feng, Qiao Ye

**Affiliations:** ^1^Department of Occupational Medicine and Toxicology, Beijing Chaoyang Hospital, Capital Medical University, Beijing, China; ^2^Clinical Center for Interstitial Lung Diseases, Beijing Institute of Respiratory Medicine, Beijing, China; ^3^Department of Occupational Diseases, The Affiliated Weihai Second Municipal Hospital of Qingdao University, Weihai, China; ^4^Department of Pathology, Peking Union Medical College Hospital (CAMS), Beijing, China

**Keywords:** granulomatosis, sarcoidosis, lung disease, case report, aluminum dust

## Abstract

**Case report:**

We present a case of a 48-year-old woman with 27 months of exposure to aluminum dust and silica owing to polishing processing. The patient was admitted to our hospital with intermittent cough and expectoration. Chest high-resolution computed tomography showed diffuse ill-defined centrilobular nodules and patchy ground-glass opacities in bilateral lungs. A video-assisted thoracoscopic surgery biopsy demonstrated multiple isolated and confluent granulomas in an otherwise normal parenchyma without malignancy or signs of infection. Elemental analysis was performed on the grinding wheel powder in the workplace using an X-ray fluorescence spectrometric analyzer, showing 72.7% of Al_2_O_3_ and 22.8% of SiO_2_ as raw materials. She was diagnosed with aluminum-associated sarcoid-like granulomatous lung disease, rather than sarcoidosis, according to occupational exposure by a multidisciplinary panel.

**Conclusion:**

Occupational aluminum dust exposure may induce pulmonary sarcoid-like granulomatosis recognized by a multidisciplinary diagnostic panel.

## Introduction

Exposure to aluminum dust may induce a wide range of pulmonary lesions in humans, including granulomatous pneumonia (1), pulmonary granulomatosis, pulmonary fibrosis (2), pulmonary alveolar proteinosis (3), and desquamative interstitial pneumonia (DIP) (4). In most reported patients, the main histological pictures were the presence of diffuse and extensive interstitial pulmonary fibrosis with variable degrees of emphysema. Despite these, aluminum-induced diffuse parenchymal disease has remained controversial owing to the relatively uncommon occurrence of interstitial lung diseases in aluminum-exposed workers (5, 6). Various environmental and occupational exposures have been related to sarcoidosis and sarcoid-like granulomatous lung diseases, which showed epithelioid granulomas that are pathologically and clinically indistinguishable from pulmonary sarcoidosis (7–9). To the best of our knowledge, granulomatous lung disease induced by aluminum dust is rare. The patients with aluminum-induced pulmonary inflammation, with lung biopsies showing granulomas, were similar to those found in sarcoidosis and chronic beryllium disease (9).

Understanding the potential role of aluminum in the development of lung granulomas in humans is limited to patient reports. Our research in medical literature led us to find 10 patients with granulomas secondary to aluminum or multiple exposures including aluminum, and the results are presented in Table 1. Two and eight patients exposed to aluminum and multiple metal exposures including aluminum, respectively, were reported. Nine of these patients had sarcoid-like granulomatosis patterns on their lung biopsies, and one patient had DIP associated with pulmonary granulomatosis.

**Table 1:** Literature review.

**No. of patients**	**Age** **(yr)**	**Sex**	**Occupation**	**Tissue sample**	**Reference**	**Location** **(country)**	**Histology**	**Elemental analysis**	**Corticosteroids** **(Y/N/NA)**	**Prognosis**
1	31	M	Welder in the aircraft industry for 5 years	Open lung biopsy	Chen et al. (1)	USA	Diffuse pulmonary granulomatosis	Aluminum	NA	NA
2	32	M	Chemist in a catalyst fabrication plant for 8 years	TBLB	De Vuyst et al. (10)	Belgium	Sarcoid-like epithelioid granulomas with Langhans-type giant cells	Aluminum, iron, silicon, aluminosilicates, rare nickel, tin, chromium, and titanium	N	16 months later, chest radiograph and lung function show no significant changes
3	37	M	Dental laboratory technician for 20 years	VATS biopsy	Brancaleone et al. (11)	Belgium	Non-caseating epitheloid granulomas	Aluminum, silicon, silicates, beryllium	Inhaled corticosteroid	2 years later, exertional dyspnea and a little cough persist. Lung function and arterial blood gas remain unchanged
4	50	F	A worker in a metal reclamation factory for 15 years	Open lung biopsy	Cai et al. (12)	China	Non-necrotizing granulomas with multinucleated giant cells	Aluminum	Oral corticosteroid	Clinical, functional, and radiological improvement
5	64	F	To polish wood furniture with sandpaper and wire brush without protection for 40 years	Lymph node biopsy	Catinon et al. (13)	France	Non-necrotizing granulomas with epithelioid cells and multinucleated giant cells	Aluminum; silicates; steel (iron, chromium, and nickel); silica; iron; calcite; titanium	NA	NA
6	33	M	A worker in the battery manufacturing industry for 7 years	VATS biopsy	Tomioka et al. (14)	Japan	Epithelioid cell granuloma	Aluminum; silicon; iron; and titanium	N	4 years later, his VC is reduced by 12.5% and DLco is induced by 12.8%. His chest HRCT shows no changes
7	46	M	A worker in an aluminum-processing factory for 6 years	VATS biopsy	Tomioka et al. (14)	Japan	Epithelioid cell granuloma	Aluminum, silicon, iron, and titanium	N	6 years later, his chest HRCT shows no changes and no respiratory symptoms
8	44	M	To brush and polish surgical instruments without any protection for 7 years	VATS biopsy	Catinon et al. (15)	France	Granuloma with multinucleated giant cells	Aluminum, silicates, silicon, iron, steel, and titanium	NA	NA
9	59	M	A worker in a company that produced refractory material for 28 years	Lung biopsy	Baur et al. (16)	Germany	Necrotizing/focally infarcted granulomas and chronic lymphoplasmacytic inflammation	Aluminum, silicon, titanium, zirconium, niobium, vanadium, and steel	NA	NA
10	57	M	As a plumber exposed to asbestos, then a worker exposed to multiple metals (not detailed the exposure time)	VATS biopsy	Blin et al. (17)	France	DIP + non-necrotizing granulomas	Aluminum, zirconium, steel	Y	1-year evaluation: clinical, functional, and radiological improvement

Video-assisted thoracoscopic surgery (VATS) biopsy; TBLB, transbronchial lung biopsy; NA, not known or not done.

The first patient with pulmonary granulomatosis associated with aluminum-containing welding fumes was reported in 1978 (1). Histological examination showed extensive interstitial granulomas composed of macrophages, foreign body giant cells, and many birefringent crystalline structures. Later, De Vuyst et al. (10) reported a patient (a chemist by profession) whose histological examination showed sarcoid-like epithelioid granulomatosis with Langhans-type giant cells. These granulomas contained dust identified by mineralogic analyses as consisting of aluminum, iron, silica, aluminosilicates, and rare occurrences of Ni, Sn, Cr, stainless steel (FeCrNi), and titanium oxides. No corticosteroids were administered, and 16 months later, chest radiograph and lung function showed no significant changes. Brancaleone et al. (11) reported a case of a dental technician who presented with non-caseating foreign body granulomas at histological examination. The bronchoalveolar lavage (BAL) lymphocytic transformation test (LTT) to beryllium nitrate detected a beryllium sensitization. Mineralogic studies showed the presence of aluminum, silica, and silicates. This patient developed lung granulomatosis most likely related to beryllium and aluminum. Cai et al. (12) reported a patient with sarcoid-like granulomatosis related to aluminum dust. High-resolution computed tomography (HRCT) showed bilateral ground-glass attenuation, patchy consolidation, extensive reticular hyperattenuating areas, and traction bronchiectasis. After 1 month of treatment with prednisone, the ground-glass attenuation decreased; the symptoms of cough, sputum, and dyspnea improved; and diffusing capacity of carbon monoxide (DLco) improved. From 2014 to 2019, five patients with granulomatosis lung disease exposed to multiple metal dust (all including aluminum) and silica were reported (13–16). Two patients were not treated with corticosteroids, while the therapy undertaken by the other three patients was not mentioned. In 2020, the first patient with DIP and pulmonary granulomatosis secondary to multiple metal exposure was reported (17). The authors concluded that DIP was associated with pulmonary granulomatosis linked to aluminum and zirconium exposure. The clinical, functional, and radiological evolution were favorable after 1 year of systemic corticosteroid treatment.

In this report, we describe a patient with pulmonary sarcoid-like granulomatosis in an aluminum polisher with clinical history, radiographic and histopathological findings, and mineralogical analyses performed on lung tissue obtained by surgical lung biopsy.

## Case description

**Figure 1:**
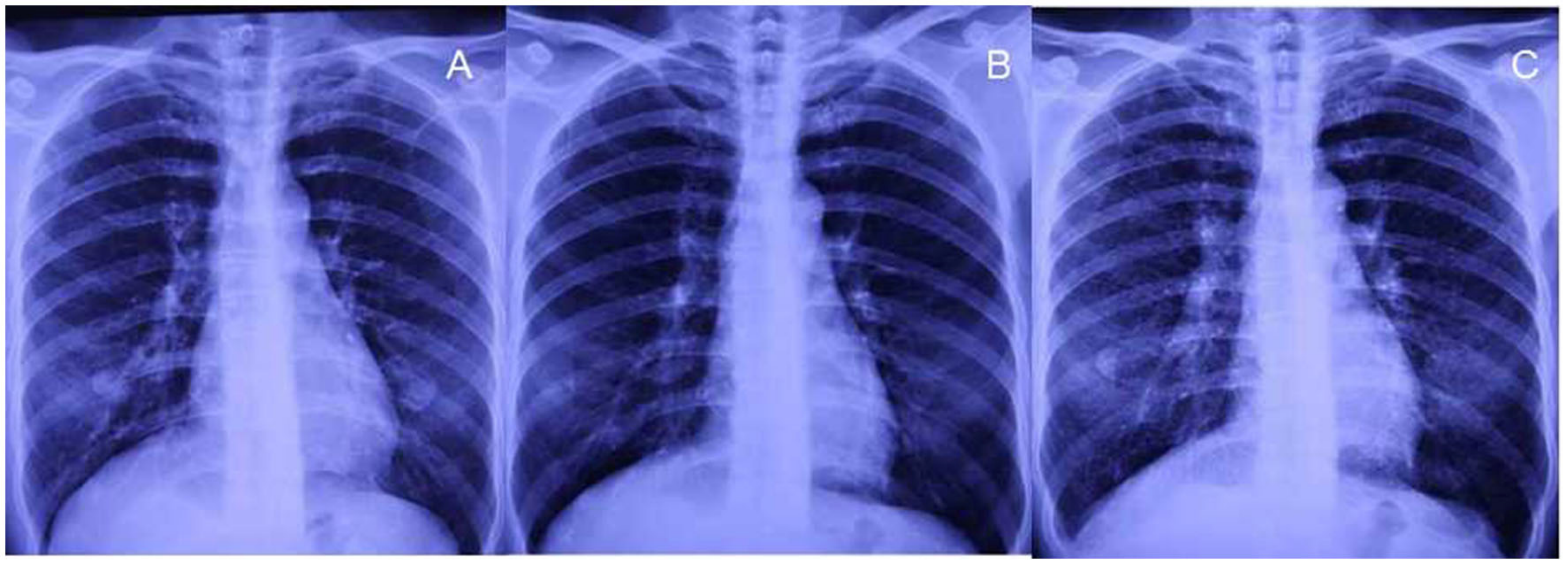
Chest radiographs before exposure and after 27 months of exposure. **(A, B)** No small nodules are observed on chest radiographs before exposure and after exposure for 15 months. **(C)** Diffuse small nodules are demonstrated on the radiograph after 27 months of exposure during work.

**Figure 2:**
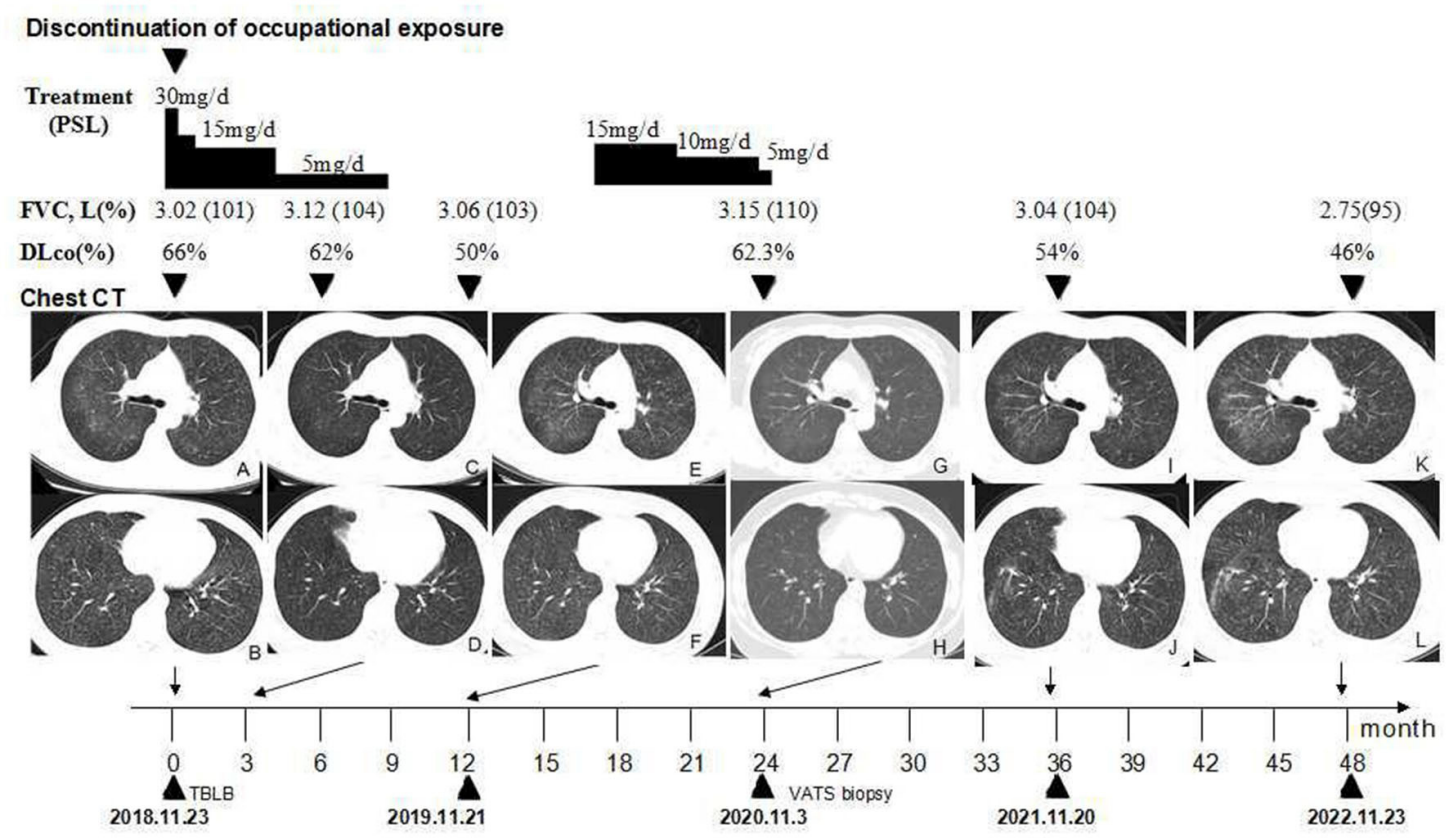
Timeline of the clinical course. **(A, B)** Chest CT showing scattered GGOs on bilateral lobular areas of ground glass attenuation and centrilobular nodules throughout the lung parenchyma before treatment. **(C, D)** GGOs are significantly reduced after the cessation of occupational exposure and using oral prednisone for 3 months. **(E, F)** GGOs and centrilobular nodules recurred after the cessation of prednisone for 3 months. **(G, H)** GGOs and centrilobular nodules significantly reduced after the second course of using oral prednisone for approximately 28 weeks. **(I–L)** Follow-up after the cessation of occupational exposure for 3 years **(I, J)** and 4 years **(K, L)**, the number of ground glass nodules in her lungs is slightly increased when compared, and fiber streak shadows are observed in the lower lobe of her right lung. CT, high-resolution computed tomography; GGOs, ground-glass opacities; PSL, prednisolone.

A 48-year-old woman presented with intermittent cough and expectoration, which she had been experiencing since May 2018. In early October 2018, the aforementioned symptoms significantly progressed with dyspnea on exertion. A chest radiograph recorded at a local clinic showed increased and disordered lung markings and scattered small patchy shadows in bilateral lungs. She was diagnosed with bronchopneumonia and was administered antibiotics; however, there was no improvement in dyspnea. On 8 October 2018, her chest radiograph of regular occupational medical check-ups at the local occupational disease hospital showed diffuse punctate and nodular shadows in the bilateral lungs (Figure 1B), while the initial chest radiograph taken on June 2016 (before work) was normal (Figure 1A). Chest CT scans revealed diffuse ill-defined centrilobular nodules, as well as patchy ground-glass opacities (GGOs) in bilateral lungs, and no pleural effusion was present (Figures 2A, B). The patient quit work and was admitted to a local occupational hospital for further evaluation.

On admission, arterial oxygen tension (PaO_2_) was 88 mmHg at rest in room air. Her routine hematological and biochemical blood tests were normal. Autoantibodies such as an antinuclear antibody, rheumatoid factor, anti-single stranded DNA antibody, anti-double stranded DNA antibody, anti-extractable nuclear antigen antibodies, and anti-neutrophil cytoplasmic antibodies were negative. At initial presentation, pulmonary function revealed a mild obstructive pattern, with forced vital capacity (FVC) of 3.02 L (101% predicted), total lung capacity (TLC) of 5.24 L (110% predicted), forced expiratory volume in 1 s (FEV1) of 2.28 L (89% predicted), FEV1/FVC ratio of 0.75, and a reduction of diffusing capacity of carbon monoxide (DLco) of 66% predicted (Figure 2). The histological findings of transbronchial lung biopsy (TBLB) revealed sarcoid-like granulomas with multinucleated giant cells, while birefringent particles were observed in the granulomas under a polarizing microscope.

The patient worked as an accountant in a restaurant when she was 19 years old for 8 years, as a bottler in a mineral water company for 4 years, and as a housewife for the next 4 years. Then she was engaged in attaching labels to fishing rods in a factory from 2007 to 2016. She had no occupational dust or fume exposure during these three early careers. From 6th July 2016 to 8th Oct 2018, the patient was engaged in polishing snowboards at a sporting products company. She worked 5–6 days per week and 8–12 h per day, using white corundum electric grinding wheels to polish the polyethylene baseplate of snowboards. It was a wet process with water-soluble cutting fluid spraying on the electric grinding wheels. The chief constituents of water-soluble cutting fluid were polyether, lauric acid, amine, and 1H-benzotriazole. She wore dust respirators during the operation. The workplace was ~2,000 m^2^ with 12 production assembly lines. There were four grinding machines with eight electric grinding wheels in every assembly line. The patient and four colleagues worked together on the same assembly line, using 30 grinding wheels each month, each weighing 10 kg. None of her 106 colleagues in the same workplace reported similar symptoms and their chest radiographs of regular occupational medical check-ups were all normal.

The level of air dust in the workplace from 2016 to 2018 was detected. Exposure concentration of time-weighted average (C_TWA_) and short-term (C_STEL_) of mixed dust in a different spot of the workplace was 1.05–8 times the occupational exposure limits (OELs) in 2016 and 2017, while the concentration of polyethylene dust was within the OELs. The maximum C_TWA_ and C_STEL_ of mixed dust were 57.5 and 55.7 mg/m^3^, respectively, exceeding the permissible concentration-time weighted average (8 mg/m^3^) by seven times. Elemental analysis was performed on the grinding wheel powder in the workplace by an X-ray fluorescence spectrometric analyzer. The assay showed that 72.7% of Al_2_O_3_ and 22.8% of SiO_2_ were raw materials. The water-soluble cutting fluid was detected. Microorganism stains and cultures, such as bacteria, acid-fast bacilli, and fungi, were negative.

The patient resigned from her job. She was diagnosed with pneumoconiosis at a local occupational hospital and received oral prednisone with a dosage of 0.5 mg/kg/day for 2 weeks. The dosage was gradually tapered to 5 mg/day for a total of 36 weeks (Figure 2). The discontinuation of occupational exposure and the first course of using corticosteroids resulted in an improvement of symptoms and a slight reduction of the multiple nodules in lung fields (Figures 2C, D). In November 2019, after the cessation of oral prednisone for 3 months, the symptoms worsened, her lung function value of DLco (Figure 2) was significantly reduced to 50%, and findings on the chest CT (Figures 2E, F) were more severe than before. She received oral prednisone again with an initial dosage of 15 mg/day from 23 April 2020. The dosage was gradually tapered to 5 mg/day (Figure 2). The respiratory symptoms were improved. She was then admitted to our hospital for further medical evaluation on 3 Nov 2020. The patient had no history of other diseases. She was a non-smoker and denied having tuberculosis, night sweats, fever, chills, or weight loss previously.

**Figure 3:**
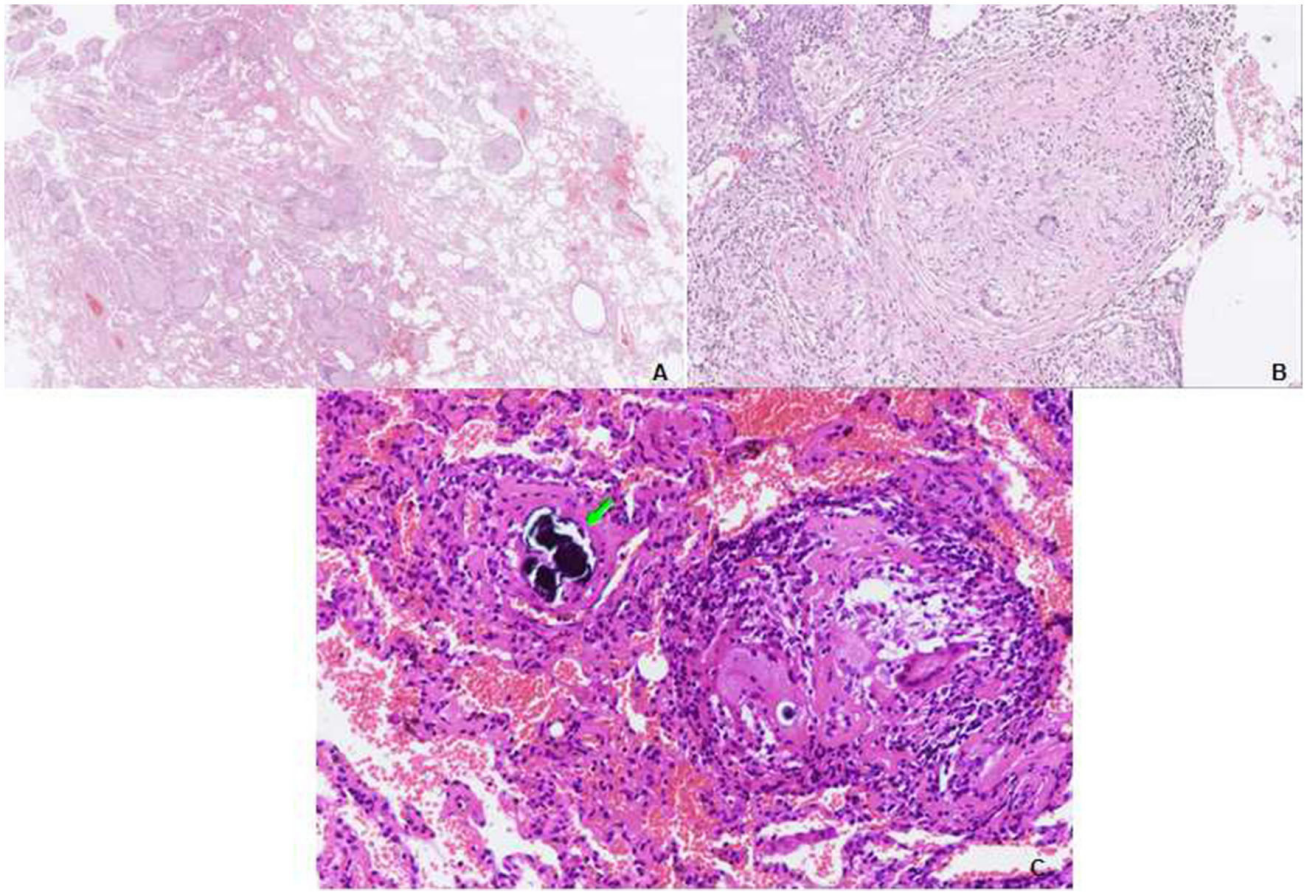
Transbronchial lung biopsy (TBLB) of the right lower lobe. **(A)** Lower power view showing the granulomas distributed in the interstitium, not in the airspace. Some of them have a centrilobular distribution, while some are distributed in the interlobular septum. A video-assisted thoracoscopic surgery (VATS) biopsy of the right lower lobe. **(B)** Many well-formed non-necrotizing granulomas are composed of epithelioid and multinucleated giant cells, with fibrous hyperplasia (hematoxylin and eosin [HE], ×40). **(C)** Multiple sarcoid-like granulomas composed of clustered epithelioid and multinucleated giant cells, a Schaumann body is observed (arrows) (HE, ×100).

Physical examination revealed chest roughness on auscultation. The remaining clinical examination was without particularity, and no extrathoracic signs or symptoms such as arthralgia, myalgia, dry eye, or dry mouth syndrome were found. The patient had no history of asthma, allergies, or any family history of respiratory disorders. Her lung function was almost improved to the initial level, with FVC of 3.15 L (110% predicted) and DLco of 62.3% predicted (Figure 2) after the second course of using prednisone in the local hospital. HRCT revealed multiple ill-defined centrilobular nodules and patchy GGOs gradually reduced with oral prednisone therapy (Figures 2G, H). Neither evidence of pleural fluid nor cardiac enlargement was noted. A video-assisted thoracoscopic surgery (VATS) biopsy of the right lower lobe was performed for further examination. The histological appearance under the microscope showed many well-formed non-necrotizing granulomas composed of epithelioid and multinucleated giant cells, mainly around the bronchi and vessels, with fibrous hyperplasia and lymphocytic inflammation (Figures 3A, B); a Schaumann body was also observed (arrows) (Figure 3C). Lightly pigmented dust macules within the interstitium were observed with adjacent focal emphysema. No classic silicotic nodules were detected. Microorganism stains and cultures, including acid-fast bacilli and fungi, were negative. In addition, 122.55 μg/g of aluminum and 455.23 μg/g of silicon were detected in the lung tissue by inductively coupled plasma mass spectrometry (ICP-MS) and inductively coupled plasma emission spectrometer (ICP-AES). No tungsten, cobalt, or beryllium was detected in both grinding wheel powder or biopsy samples.

Based on occupational dust exposure combined with clinical, radiological, and histological findings of sarcoid-like granulomatosis, the patient was diagnosed with aluminum-associated sarcoid-like granulomatous lung disease by a multidisciplinary panel, rather than sarcoidosis. Upon admission to our hospital in November 2020, the respiratory symptoms, chest HRCT scans, and lung function of DLco improved; therefore, the second course of prednisone therapy lasted for a total of ~ 28 weeks. She was administered oral N-acetylcysteine and inhalational corticosteroids. After discharge from our hospital, she was followed up regularly in a local occupational hospital. During the 36-month follow-up observational period, her chest CT (Figures 2I, J) revealed that GGOs slightly increased, and fiber streak shadows were observed in the lower lobe of the right lung, as well as the value of DLco reduced to 54%; however, her respiratory symptoms were stable. A 48-month follow-up revealed that her symptoms of dyspnea on exertion gradually worsened, while GGOs (Figures 2K, L) gradually increased and DLco reduced to 46%. The patient gained weight significantly after oral glucocorticoid treatment. After stopping the glucocorticoid, the shortness of breath was not obvious in the resting state, so she resisted the use of glucocorticoid therapy again.

This study was approved by the Medical Ethical Committee and the patient provided informed consent for the publication of the case.

## Discussion

The differential diagnosis of sarcoid-like granulomatosis induced by aluminum is challenging. First, the disease is not well-established and easy to be misdiagnosed. Second, it may be difficult to distinguish from sarcoidosis, especially by the pathological manifestations. Third, a multidisciplinary panel including occupational specialists may be essential for enhancing the accuracy of the diagnosis. A variety of infectious, occupational, and environmental factors have been implicated in sarcoid-like granulomatous lung disease (7, 8, 18). As infection is a common cause of pulmonary granulomas, infectious lung diseases must be excluded. Infectious diseases may reasonably be excluded for the absence of serologic and bacteriologic abnormalities.

Silicon was detected in both grinding wheel powder and biopsy samples. Under a polarizing microscope, several birefringence particles can be observed in the granulomas of TBLB tissues. Silicosis is common in workers who produce corundum grinding wheels and has an exposure–response relationship. The cumulative dose of silica exposure, which is respirable dust concentration multiplied by crystalline silica content and exposure duration, is considered the most important factor in the development of silicosis (19, 20). This patient was a grinder and the radiograph showed bilateral diffuse small nodules after working for only 2 years and 3 months. The elemental analysis showed a relatively low content of free silica (22.8% of SiO_2_) in the raw grinding wheel powder. The surgical biopsy demonstrated that multiple sarcoid-like granulomas were composed of clustered epithelioid and multinucleated giant cells, while the silica nodules were mainly composed of dust-laden hyalinized collagen, and pathologic findings of aluminous showed diffuse interstitial fibrosis with emphysema. Recent case reports have postulated that sarcoid-like granulomatous lung disease can also be induced by silicates (21, 22). Different epidemiological studies have demonstrated a higher risk of sarcoidosis among persons occupationally exposed to silica (23). Regarding the role of aluminum combined with silicon or silicates in sarcoid-like granulomatous lung disease pathogenesis, no randomized control trial (RCT) studies other than case reports exist. However, in the present case report, the content of Al_2_O_3_ was much higher than that of SiO_2_ in the raw material; therefore, we diagnosed the patient with aluminous-associated sarcoid-like granulomatous lung disease.

The diagnosis of sarcoidosis is based on the exclusion of other granulomatous lung diseases (24, 25). Recent epidemiologic studies have revealed a potential correlation between occupational exposure and the disease (26). Based on occupational dust exposure and initial transbronchial lung biopsy pathology, we suspected that exposure to “grinding wheel powder” resulting from her occupational history might be implicated in the development of granulomatous pulmonary disease. With elemental analysis, the X-ray fluorescence spectrum of the grinding wheel powder yielded discrete peaks for aluminum. Meanwhile, high amounts of aluminum were detected in lung tissue using ICP-MS.

The exact mechanism that leads aluminum to induce these sarcoid-like granulomas is unclear. The metal elements may directly act as antigens to stimulate the immune system to cause sarcoidosis. The antigens may interact with the immune system to cause its dysregulation, which was involved in the formation of sarcoid (8). Immunoreactivity to metal elements had been found only in patients with sarcoidosis using a lymphoid proliferation test, suggesting that in addition to beryllium, aluminum may also be a possible stimulated antigen triggering an immune response. Even if the patient stopped exposure, the aluminum deposited in the lung may be a persistent stimulated antigen triggering an immune response. We reviewed the medical literature works and found 10 patients with granulomas secondary to aluminum or multiple exposures including aluminum. Six cases were followed up and the follow-up time spanned 1 year to 6 years. Among these cases, one patient's VC was reduced by 12.5% and DLco was induced by 12.8%, while his chest HRCT showed no changes after stopping exposure for 4 years (14). Four cases had no information of follow-up.

Pulmonary granulomatosis caused by aluminum dust or multiple other dust types (including aluminum) is an individual heterogeneous disease. The susceptible individuals may develop this disease even in a short time and on a relatively small amount of aluminum exposure. For workers exposed to aluminum dust, if the onset, symptoms, and imaging are not consistent with the characteristics of aluminum pneumoconiosis, a lung biopsy should be performed. When the pathology of lung biopsy shows sarcoid-like granulomas, oral glucocorticoid may be beneficial. The dosage and duration of glucocorticoids are still controversial. It may depend on the willingness of the patient and response to the medications. This patient took prednisone for a total of 15 months. Pulmonary symptoms, CT scans, and lung function values were improved after prednisone therapy; however, the disease recurred after the cessation of prednisone. Further insights concerning the relationship of aluminum exposure to the development of granulomatous lung disease may have a major impact on the prevention and treatment of this enigmatic disease.

## Conclusion

Aluminum-associated sarcoid-like granulomatous lung disease is rarely diagnosed. This report described the case of a patient who had suffered from extensive occupational inhalation of aluminum dust. The diagnosis was based on occupational dust exposure combined with clinical-radiological-histological findings. The findings of the present case support the association between aluminum exposure and sarcoid-like granulomatous lung disease, and the emergence of these diseases should be taken into account in the clinical course of aluminum dust exposure.

## Data availability statement

The original contributions presented in the study are included in the article/supplementary material, further inquiries can be directed to the corresponding author.

## Ethics statement

Written informed consent was obtained from the individual(s) for the publication of any potentially identifiable images or data included in this article.

## Author contributions

QY designed the study. XD and LS collected and analyzed the patient data and play an equally important role in this manuscript. LS followed up on the patient data and XD wrote the manuscript. RF performed the histological examination of the lung tissue. All authors contributed to the article and approved the submitted version.
